# The Earth's Lithosphere Inspires Materials Design

**DOI:** 10.1002/adma.202005473

**Published:** 2020-12-09

**Authors:** Yan Beygelzimer, Roman Kulagin, Peter Fratzl, Yuri Estrin

**Affiliations:** ^1^ Donetsk Institute for Physics and Engineering named after A.A. Galkin National Academy of Sciences of Ukraine Nauki Avenue, 46 Kyiv 03028 Ukraine; ^2^ Institute of Nanotechnology Karlsruhe Institute of Technology Hermann‐von‐Helmholtz‐Platz 1 Eggenstein‐Leopoldshafen 76344 Germany; ^3^ Max Planck Institute for Colloids and Interfaces Am Mühlenberg 1, OT Golm Potsdam 14476 Germany; ^4^ Department of Materials Science and Engineering Monash University 22 Alliance Lane Clayton 3800 Australia; ^5^ Department of Mechanical Engineering The University of Western Australia 35 Stirling Highway Crawley 6009 Australia

**Keywords:** architectured materials, biomimetics, lithomimetics, self‐organization, severe plastic deformation

## Abstract

Structural patterns found in living organisms have long been inspiring biomimetic materials design. Here, it is suggested that a rich palette of patterns occurring in inanimate Nature, and especially in the Earth's lithosphere, could be not less inspirational for design of novel architectured materials. This materials design paradigm is referred to as lithomimetics and it is demonstrated that some of the patterns found in the lithosphere can be emulated by established processes of severe plastic deformation. This opens up interesting avenues for materials design in which potentially promising structural patterns are borrowed from the lithosphere's repository. The key aim here is to promulgate the “lithomimetics” paradigm as a promising approach to developing novel architectured materials.

## Graphical abstract

A novel lithomimetic approach is proposed, which entails mimicking structures and processes occurring in the Earth's lithosphere to produce man‐made advanced materials. Severe plastic deformation is considered as a viable vehicle for that.

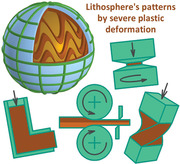

## 1 Introduction

In his programmatic treatise on structural stability and morphogenesis, René Thom noted that “for centuries the form of living beings has been an object of study by biologists, while the morphology of inert matter seems only accidentally to have excited the interest of physicochemists.”^[^ ^1^ ^]^ In relation to design of new materials, this statement is certainly true to the present day. While biomimetics, broadly understood not only as emulation of biological structures but also as a set of guiding principles for design of engineering materials, has long been motivating materials scientists, a treasure‐trove of inanimate Nature has been largely overlooked. We would like to draw the attention of the materials research community to a rich gamut of formations that emerge during processes occurring in the inanimate Earth's lithosphere as a potential source of inspiration for design of materials. By analogy with biomimetics, yet in an antonymic sense, we dub this approach lithomimetics. The lithomimetics paradigm implies that processes that occur in the Earth's crust and the patterns they produce can enrich the toolbox used in design of novel materials—again not necessarily offering direct blueprints, but rather providing hints leading to promising material architectures.

Architectured (or architected) materials, also referred to as archimats, have recently come to the fore as a new, special class of materials whose inner make‐up represents an additional degree of freedom in engineering design for advanced applications.^[^ ^2^ ^]^ According to a definition going back to M.F. Ashby,^[^ ^3^ ^]^ an architectured material comprises other materials in such a way that its properties are largely defined by the geometry and mutual arrangement of the constituents. This definition embraces both natural and engineered man‐made materials. It encompasses cellular materials, metamaterials, weaves, fiber mats, and many other types of composites. The burgeoning field of architectured materials has been advanced by the seminal work of several research groups.^[^ ^3^, ^4^ ^]^ A key feature borrowed by biomimetics from living organisms and biological materials, such as bones and armor of animals, mollusc shells, insect wings, parts of plants, etc.,^[^ ^5^, ^6^, ^7^ ^]^ is their multiscale hierarchical structure. The excellent properties of these natural structures owe to a long evolutionary process, in which mutations led to a great variety of forms, while natural selection manifested those that provide high strength, light weight, fracture toughness, and resistance to cyclic loading. It is thus understandable that researchers turned their interest to such structures first, and that is why many archimats developed so far are bioinspired.

**Figure 1:**
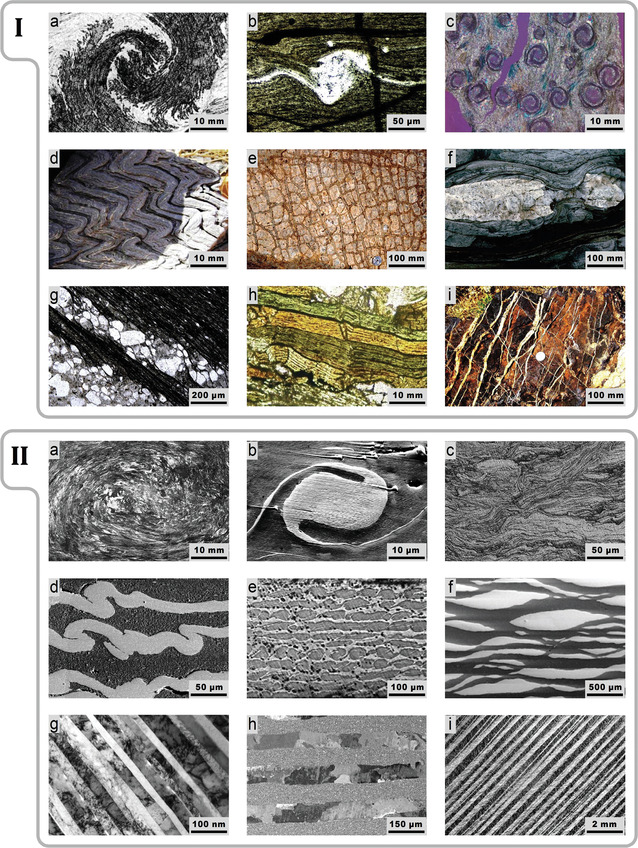
I,II) Examples of geological formations (I) and self‐organized patterns of archimats processed by SPD techniques (II): I‐a) vortex structure,^[^ ^8^ ^]^ I‐b) delta microstructure of olivine,^[^ ^10^ ^]^ I‐c) spiral garnet,^[^ ^8^ ^]^ I‐d) chevron‐like fold,^[^ ^10^ ^]^ I‐e) chocolate tablet boudinage,^[^ ^11^ ^]^ I‐f) boudinage with quartz between the boudins,^[^ ^11^ ^]^ I‐g) cleavage refraction,^[^ ^10^ ^]^ I‐h) kink bands,^[^ ^10^ ^]^ and I‐i) quartz vein network.^[^ ^10^ ^]^ The scale bars are added here based on the information provided in the original publications for images from refs. ^[^ ^8^, ^10^, ^11^ ^]^. II‐a) Vortex (Bulat‐type) structure, Al, twist extrusion,^[^ ^12^ ^]^ II‐b) delta structure, Al/Cu, high‐pressure torsion (HPT),^[^ ^22^ ^]^ II‐c) vortex‐like cleavage, Al/Ni, HPT,^[^ ^13^ ^]^ II‐d) folds, Al/Ni, HPT,^[^ ^13^ ^]^ II‐e) boudinage‐like pattern, Al/Cu, HPT,^[^ ^22^ ^]^ II‐f) pinch and swell pattern, Al/Ti, accumulated roll bonding (ARB),^[^ ^14^ ^]^ II‐g) multilayered cleavage, Cu/V, ARB,^[^ ^15^ ^]^ II‐h) multilayers, Al99.5%/Al99.9%, ARB,^[^ ^16^ ^]^ and II‐i) shear bands, Al, equal‐channel angular pressing.^[^ ^17^ ^]^ I‐a,c) Reproduced with permission.^[^ ^8^ ^]^ Copyright 2015, Elsevier. I‐b,d,g–i) Reproduced with permission.^[^ ^10^ ^]^ Copyright 2015, Elsevier. I‐e,f) Reproduced with permission.^[^ ^11^ ^]^ Copyright 2010, Cambridge University Press. II‐a) Reproduced with permission.^[^ ^12^ ^]^ Copyright 2017, Wiley‐VCH. II‐b,e) Reproduced with permission.^[^ ^22^ ^]^ Copyright 2019, Wiley‐VCH. II‐c,d) Reproduced with permission.^[^ ^13^ ^]^ Copyright 2018, Elsevier. II‐f) Reproduced under the terms of the CC‐BY Creative Commons Attribution 4.0 International license (https://creativecommons.org/licenses/by/4.0).^[^ ^14^ ^]^ Copyright 2016, The Authors, published by MDPI. II‐g) Reproduced under the terms of the CC‐BY Creative Commons Attribution 4.0 International license (https://creativecommons.org/licenses/by/4.0).^[^ ^15^ ^]^ Copyright 2017, The Authors, published by Springer Nature. II‐h) Reproduced with permission.^[^ ^16^ ^]^ Copyright 2016, Elsevier, II‐i) Reproduced under the terms of the CC‐BY Creative Commons Attribution 4.0 International license (https://creativecommons.org/licenses/by/4.0).^[^ ^17^ ^]^ Copyright 2019, Acta Materialia Inc., published by Elsevier Ltd.

We argue that in designing structural and functional architectured materials we may learn a lot from inanimate Nature. The Earth's lithosphere is a particularly rich depository of structural patterns.^[^ ^8^, ^9^, ^10^, ^11^ ^]^ Many of them have emerged as a result of transformations induced in rocks by deformation under high pressure. Examples of such patterns are shown in **Figure** **1**I.

We believe that these patterns are of interest, if only because they demonstrate that deformation‐induced transitions between the various structural forms of materials are possible without human interference. According to the philosophy of René Thom, one can expect a profound similarity between the geometrical principles governing shape development in animate and inanimate Nature.^[^ ^1^ ^]^ However, in contrast to the living world, the development of patterns in inanimate Nature is not subjected to a selection process. This has interesting consequences: on the one hand, due to lack of a biological adaptation mechanism, the properties associated with a pattern formed are not necessarily advantageous for any given function. On the other hand, physicochemical self‐organization in inanimate Nature, for example, in the evolving Earth's crust, brings about a broad variety of structural patterns. They represent a rich “atlas” of potential patterns with mostly unknown physical and mechanical properties to choose from. This collection of patterns has good reasons for being more diverse than patterns formed in the living world, as their emergence is not constrained by the limitations of natural selection and biological synthesis ruling out high temperatures and pressures. Besides, the mechanisms of morphogenesis in inanimate Nature governed by physicochemical principles may be easier to emulate than the more intricate biological processes carried out by cells.^[^ ^18^ ^]^ What distinguishes the structural patterns generated by morphogenesis in the lithosphere is that some of the underlying mechanisms can be reproduced in a laboratory and used for the design and manufacture of archimats. That is why our focus here is on the lithosphere as a repository of potentially useful structures.

## 2 The Lithomimetics Paradigm

Parallels between structural patterns formed in the lithosphere and those produced artificially become obvious when the structures presented in Figure 1I are compared with a collection of patterns occurring in materials processed by special metal working techniques collectively known as severe plastic deformation (SPD),^[^ ^19^ ^]^ see Figure 1II. These similarities stem from the mentioned commonality of the thermodynamic and geometric principles that govern patterning—despite the fundamental differences in the deformation mechanisms for metals and rocks. The criteria by which patterns emerge in SPD experiments may be different from those in the Earth's crust, but the resulting patterns will have a lot in common. The possibility to replicate the salient features of the geological patterns in synthetic materials establishes a basis for the lithomimetics paradigm we wish to publicize.

First, we discuss the patterns found in the lithosphere without going in the detail of the nomenclature of structural geology or into the geographic location of the patterns presented; we simply refer the reader to the original publications. What matters is the type of a structure and the kind of process that may be responsible for its emergence. In assessing these geological structures and the mechanisms of their formation, materials scientists can build upon the vast knowledge accumulated in structural geology.^[^ ^8^, ^9^, ^10^, ^11^ ^]^ Interesting rock morphologies are exemplified by various vortex‐like patterns in Figure 1I‐a–c. The defining conditions for the generation of such structures are the occurrence of shear deformation under pressure, a gradient of strain or stress, and the presence of fragments blocking laminar deformation.^[^ ^8^, ^9^, ^10^ ^]^ In some cases, the genesis of these structures and the processes that led to their formation can be identified based on their morphology. Thus, chevron folds presented in Figure 1I‐d can be traced back to the conditions when a stack of alternating layers of soft and hard materials, for example, sandstone and shale, are compressed at relatively low confining pressures. Concurrent shear of this stack of layers in the same direction produces a tilt seen in the figure. The origins of boudinage morphologies (Figure 1I‐e,f) formed in geological rocks can be associated with tensile deformation causing their fracturing accompanied with filling of cracks with soft matter under pressure.^[^ ^9^ ^]^ A variety of layered architectures, such as cleavage (Figure 1I‐g) or multilayer structures (Figure 1I‐h), represent a class of patterns in which shear and/or tensile stresses lead to interesting effects due to a synergistic combination of the properties of the different layers.^[^ ^9^, ^10^ ^]^ An indicator, or marker, of shear sensitivity of metamorphic processes is a vein‐type structure (Figure 1I‐i) that typically forms orthogonally to the maximum instantaneous extension axis.^[^ ^9^, ^10^ ^]^

The aesthetic appeal of the patterns in Figure 1I and the very fact that they emerge as a result of geological processes does not necessarily imply that the mechanical properties of such formations are particularly favorable and thus worthy of being emulated in engineering materials. However, we believe that they do hold potential and deserve being explored as possible prototypes of archimats. One of the reasons for this expectation is that the thermodynamic principle which governs the formation of geological patterns is that of the maximum rate of dissipation of mechanical energy.^[^ ^20^, ^21^ ^]^ It states that the actual power of dissipation corresponding to a given deformation rate is never less than a fictitious power of dissipation calculated from the actual deformation rate and a stress state at or below the yield limit. It follows that the lithospheric patterns should be efficient with regard to dissipation of the work of external forces, so that their transformation under the action of these forces is hindered. It can thus be expected that fracture of an architrectured material replicating this kind of patterns should also involve large energy dissipation. That is to say, emulating such patterns in structural materials would provide them with high fracture toughness.

Now we turn to SPD processes^[^ ^19^ ^]^ that can be employed to generate lithosphere‐inspired patterns in metallic materials. Indeed, a key feature of SPD processing—a combination of giant simple shear deformation with high hydrostatic pressure—is akin to the conditions leading to the metamorphic structure formation in the lithosphere. Thus, SPD techniques provide a possibility to translate at least some mechanisms governing the processes in the lithosphere to a laboratory environment. As an example, metal–metal composites of a special kind with a favorable lithomimetic inner architecture can be obtained in this way. The length scale at which such architectures can be realized in SPD‐processed materials is intermediate between the microscale and that defined by the sample dimensions. An added benefit of this type of processing is that the constituents of the archimats thus obtained possess an ultrafine structure in the sub‐micrometer range and, as a result, exhibit exceptionally good mechanical properties, notably greatly enhanced strength, often combined with improved ductility.^[^ ^22^, ^23^, ^24^, ^25^, ^26^ ^]^ This propensity for extreme microstructure refinement in bulk, full‐density materials is a great benefit of SPD‐synthesized archimats over those obtained, for example, by additive manufacturing.

The ability of SPD methods to produce self‐organized structures has been demonstrated in recent publications,^[^ ^22^, ^23^, ^24^, ^27^ ^]^ as is illustrated by the images of various systems processed by these techniques in Figure 1II. The striking similarity of the material architectures captured in these images with the metamorphic formations collated in Figure 1I is quite remarkable and evocative.

The vortex (Bulat‐like) architecture in Figure 1II‐a is a result of the vortex flow of aluminium under twist extrusion which produced a “frozen” grain texture at macroscale. The structures seen in Figure 1II‐b–e represent examples of the patterns formed under the deformation of multilayered samples by high‐pressure torsion (HPT)—a process in which a penny‐shaped specimen undergoes torsional deformation in a Bridgman die under a gigapascal‐scale pressure.^[^ ^19^, ^23^ ^]^ Instability of the harder layers may get localized either as delta structure (Figure 1II‐b) or folds (Figure 1II‐d). If multilayered structures are made from materials with a strong contrast in the yield strength, delamination between the layers may occur at sufficiently large strains, thus forming vortex‐like cleavage structures (Figure 1II‐c). Furthermore, subdivision of the layers by vortices leads to the formation of boudinage‐like structures (Figure 1II‐e). Material architectures presented in Figure 1II‐f–h represent the variants of self‐organization during an accumulative roll bonding (ARB). (ARB is a process involving multiple steps of rolling, cutting the sheets in halves, re‐stacking them, and repeat rolling.) Pure shear of a multilayered material may lead to the occurrence of structures of the pinch‐and‐swell type (Figure 1II‐f), with a thinning of the layers down to the grain size (Figure 1II‐g), or to the formation of a laminate comprising coarse‐grained and ultrafine‐grained layers (Figure 1II‐h). An example of a uniform sample developing non‐homogeneous patterns under plastic flow at the scale of individual grains is shown in Figure 1II‐i. Stratification to a laminate consisting of alternating layers with coarse‐ and ultrafine‐grained structure stems from self‐organized shear localization during equal‐channel angular pressing.^[^ ^17^ ^]^

Some of the promising candidate architectures found in geological formations involve delta and vortex structures (Figure 1I‐a–c). They also occur in Bulat and Damascene steels known for their superb mechanical properties.^[^ ^28^ ^]^ To mimic such structures in novel archimats, the SPD technique of HPT or twist extrusion can be used (Figure 1II‐a–c).^[^ ^12^, ^13^, ^27^ ^]^ Structures inspired by geological folds (Figure 1I‐d) are expected to obtain similar properties owing to the blockage of crack propagation through developed interfaces between the layers. The parallels between the materials architectures seen in Figure 1II and those collated in Figure 1I are very telling, indeed.

Chocolate tablet boudinage (Figure 1I‐e) and quartz vein structures (Figure 1I‐i) motivated the “artificial crystal” design we proposed earlier. In such “artificial crystals”, thin layers of a relatively soft metal mediate plastic strain that is imposed on a hard sub‐micrometer‐structured archimat at extremely high stresses. We produced such structures by HPT of stacks of alternating copper and aluminium layers (Figure 1II‐e).^[^ ^13^ ^]^ HPT based on extreme shear deformation by torsion under a hydrostatic pressure amounting to severalfold the yield strength of the material resembles the processes in the Earth's crust,^[^ ^29^ ^]^ for example under conditions when sedimented layers are deformed in shear under compressive stress normal to the layers.^[^ ^9^ ^]^ The “artificial crystal” architecture of the archimat thus produced was shown to reduce its sensitivity to tensile overloads and to inhibit its catastrophic failure. The outstanding properties of archimats obtained by employing a process that emulates a metamorphic process in the lithosphere are motivating, as they exemplify how lithomimetics may work for a broader range of structural patterns.

The geological structures of the cleavage type (Figure 1I‐g) suggest a design of archimats containing soft metal interlayers at the sites where separation of the matrix occurs. These interlayers will be efficient in arresting small cracks and blocking their propagation. A way to obtain materials with this kind of architecture is by employing HPT of stacks of layers with contrasting hardness, Figure 1II‐c, or by using ARB, Figure 1II‐g.^[^ ^13^, ^15^ ^]^ Multilayer formations found in structural geology (Figure 1I‐h) are also a common motif in metallic composites. Lamellar structures with strong cohesion between the layers can be obtained by ARB, often at room temperature,^[^ ^16^, ^24^ ^]^ or by equal channel angular pressing, Figure 1II‐i (arguably the most popular SPD technique in which a billet is repeatedly pressed through a die with an angular channel).^[^ ^17^, ^19^ ^]^ Processing at ambient temperature makes it possible to avoid the formation of undesirable brittle intermetallic phases and enables good cohesion at the interface. Examples of such laminates are seen in Figure 1II‐f–h.

The above juxtaposition of patterns found in the lithosphere and their counterparts obtained by SPD processing methods shows that for each geological structure presented, there is a look‐alike pattern obtained by SPD. We see this as a convincing demonstration of the feasibility of imitating the processes occurring in the Earth's crust by readily available metal forming techniques.

## 3 Similarities and Differences between Biomimetics and Lithomimetics

When creating bioinspired materials, one borrows an idea of a structure from living Nature, and implements it in an artificial material through specific actions using such techniques as lithography, additive manufacturing, welding, stamping, etc. What we suggest here is to use, in creating archimats with self‐organized inner architecture, principal processes borrowed from inanimate Nature. As a guiding prototype, we consider self‐organization of structures in the Earth's crust under deformation, pressure, and heat (all being non‐specific actions). Empirical observations reveal an astoundingly wide range of various structures that emerged in this way. Among them are purely mechanistically generated structures, such as folds of various kinds, boudins, shear bands, cleavages, etc., as well as patterns emerging as a result of physicochemical transformations, including cellular and vortex‐like ones. Some of these structures may arouse the interest of researchers working in various areas of materials science, as their diverse topologies offer promising avenues for obtaining new physical properties. The extreme diversity of patterns is a function of the great variability of strain, pressure, and heat effects occurring in the Earth's crust.

While the same principal mechanisms of self‐organization govern the morphogenesis in animate and inanimate Nature (and while a unified mathematical description is possible),^[^ ^30^ ^]^ the methodology of archimat design based on analogies with these two distinct realms of Nature ought to be different. In biomimetics, we utilize the designs of animate Nature, that is, the materials it has developed in the course of evolution, as prototypes for certain biologically relevant functions to be fulfilled by an artificial archimat. As distinct from biomimetics, lithomimetics seeks the processes that lead to promising geology‐inspired structures of prospective archimats. We do not see these structures as ready‐made “blueprints” for engineering materials, though. Rather, they are tips geology gives us, and it will certainly take a great deal of scientific effort to assess their suitability as models worth emulating. The above examples of lithosphere‐inspired archimats whose structure was formed by nonspecific external conditions, such as pressure and deformation, give promise that research in this area would be of potential value. The knowledge accumulated in biomimetics is very substantial. A lot needs to be done for lithomimetics to become equally successful. The parallels between lithomorphic architectures and the structures produced by severe plastic deformation (notably HPT) are a useful indication of the sort of processing techniques that may provide a pathway to lithomimetics.^[^ ^23^ ^]^ We should also like to mention that the richness of the beautiful structures that evolved in inanimate Nature is not limited to the geological formations shown in Figure 1I. Further patterns, for example, Liesegang rings, columnar quasihexagonal patterns, minimal surfaces in rocks (“Earth's bubbles”, to paraphrase Shakespeare's metaphor),^[^ ^31^ ^]^ an amazing variety of sandstone layers in the Grand Canyon (Arizona, USA), and many others, can also serve as inspiring examples of material architectures that may be worth copying in novel archimats.

The two types of architectures found in the treasure chest of Nature—both animate and inanimate—are products of an extremely long development. In animate Nature, evolution occurs owing to two factors: mutations producing new variants of species and natural selection proliferating the best of them. In geological morphogenesis, structures of potential interest develop in the lithosphere by self‐organization occurring over geological times. Mimicking these architectures in artificial, human‐made engineering structures is a final step that in a sense can also be regarded as a natural one, as humans are part of Nature, too. However, to the backdrop of natural evolution that took millions and millions of years, artificial evolution underlying engineering design appears to be almost instantaneous. In that process, Nature does not provide ready solutions, yet it offers some hints that may lead to success.

## 4 Where Do We Go from Here?

Why do we think it is worthwhile to invest in the development of the field of lithomimetics if it does not warrant an immediate return in terms of exceptional material properties? Here are some reasons for that. First, the huge variety of patterns that have emerged in the Earth during its development is too attractive to pass by without looking into the potential they may offer. Second, the above examples demonstrate the precedence of good mechanical properties (including high strength and enhanced fracture resistance) exhibited by at least some of the lithomorphic architectures that have already been replicated in metallic materials, for example, in laminated copper and aluminum composites produced by SPD. Third, the simplicity of the SPD processing as a means of obtaining lithomimetic structures is undoubtedly their forte when it comes to manufacturing metallic archimats. We believe this type of processing relying on self‐organization may be preferable to 3D printing or assembling of archimats from prefabricated parts. Fourth, the use of SPD techniques, which essentially are variants of more traditional metal working processes, enables production of dense parts with good cohesion between their constituents. It also offers a double benefit of architecturing the end material while at the same time strengthening its constituents by extreme grain refinement, cf. Chapter 8 of the cited monograph.^[^ ^2^ ^]^

The new research direction we are proposing provides conditions for cross‐fertilization of materials science and metal working on one side and structural geology on the other. It is to be seen in the tradition of synergy between mathematical and physicochemical investigations of irreversible deformation founded in the classical works of P. Bridgman, M. Biot, and T. von Kàrmàn. Each of these outstanding researchers who worked in the area of plasticity turned to studies of the formation of geological structures. However, they did not set the goal of emulating such structures or use the underlying processes in design of engineered materials, which is the target of the lithomimetics approach presented here. Involving SPD technology in these studies would give this research a new twist enabling fabrication of lithomimetic structures with metallic, ceramic, and polymeric materials. To that end, one would have to find compositions and simple initial structures (such as layers of different thickness, matrix materials with inclusions, fibers, etc.) and unravel the mechanisms of their self‐organization towards desired final architectures induced by deformation. Studying self‐organization achieved by using a combination of different SPD processes would provide further possibilities of producing interesting material architectures. Self‐organization involving deformation‐induced physicochemical transformations is a further promising area of research. This will require the use of deformation processing in conjunction with thermal treatment. At a first stage, the mass transport dramatically enhanced by SPD will enable close contact between the different constituent materials. At a second stage, these substances will react chemically by diffusion, which may lead to spatial patterning, for example, of the Turing type. Among the analytical tools available for studying Turing instabilities, which generate self‐organized patterns in rocks, the coupled reaction–diffusion–deformation equations proposed by Hobbs and Ord appear to be particularly suitable.^[^ ^31^ ^]^ Their approach is also believed to be helpful in getting insights in the metamorphic processes behind the patterns observed, thus facilitating lithomimetic materials design. Of special interest is incorporation of deformation‐induced chemical reactions (“deformation‐induced synthesis” of materials)^[^ ^22^, ^23^ ^]^ into this analysis in analogy to the analysis of metamorphic rocks.^[^ ^31^ ^]^ Coupled mechanical–chemical modelling approaches developed to describe chemical zoning in minerals can also be adopted for lithomimetic design.^[^ ^32^ ^]^

The lithosphere offers us an immense variety of structural patterns, which can be tapped into when designing architectured engineering materials. Unlike the animate Nature, the lithosphere does not have the benefit of adaptation through interplay of mutations and natural selection, but, on the other hand, it is not constrained by the narrow corset which biological synthesis puts on living matter. It may thus offer an even broader variety of patterns stimulating materials design. Most of the examples outlined in this note are based on severe plastic deformation of mostly inorganic materials, such as metals and alloys, which is regarded as a viable pathway to producing material architectures suggested by the geological formations. A requisite basis for a future lithomimetic approach to materials development is a closer collaboration between geoscience and materials science. Indeed, the knowledge of the genesis of complex lithospheric structures accumulated in geoscience can enable materials scientists to find the right processing routes leading to targeted materials design. Conversely, the profound understanding of the deformation processes the discipline of materials science has established can assist geoscience in interpreting geological formations. In this endeavor, the use of severe plastic deformation in laboratory‐scale experiments as a testbed for replicating metamorphism in the lithosphere appears to be an interesting and promising perspective. Understanding and, eventually, imitating the processes underlying pattern formation in the lithosphere is at the core of the lithomimetic approach as we understand it. Whether these patterns furnish the best mechanical or functional properties is subject to investigation, and the result for a particular pattern may well be negative. However, while the desired outcome cannot be guaranteed, the prospects of having success through digging into this rich arsenal of structures should motivate research in the nascent area of lithomimetics.

## Conflict of Interest

The authors declare no conflict of interest.
